# Histological studies on the relationship between the low seed set and abnormal embryo sacs in sweet potato, *Ipomoea batatas* (L.) Lam.

**DOI:** 10.1270/jsbbs.23022

**Published:** 2023-09-09

**Authors:** Tatsuro Murata

**Affiliations:** 1 School of Agriculture, Tokai University, 871-12 Sugidou, Mashiki-machi, Kamimashiki-gun, Kumamoto 861-2205, Japan

**Keywords:** sweet potato, *Ipomoea batatas*, *Ipomoea trifida*, low seed set, abnormal embryo sac

## Abstract

This study aimed to investigate the relationship between low seed set and abnormal embryo sacs lacking normal female organs, such as one egg cell, two assistant cells, and two polar nuclei, in *Ipomoea trifida*, which is closely related to sweet potato, and sweet potato cultivars and lines, through histological analysis of their ovaries on flowering day. Ovaries of diploid, tetraploid, and hexaploid lines of *I. trifida* each had four ovules, except for some hexaploid lines with five or six ovules. Almost all sweet potato cultivars and lines had four ovules per ovary, although some sib-cross lines had two or three ovules. The number of ovules per ovary did not have direct effects on low seed set. The frequency of abnormal embryo sac increased with polyploidy in *I. trifida*. However, it varied among different sweet potato cultivars and lines. Moreover, the variation in abnormal embryo sacs occurred at an earlier stage of gametogenesis (type A) in the tetraploid and hexaploid plants of *I. trifida* and sweet potato cultivars and lines. These findings suggest that the high frequency of abnormal embryo sacs is a primary cause of low seed set in sweet potato and that it is closely related to the decline in seed propagation that occurs in the evolution process of sweet potato.

## Introduction

Most sweet potato cultivars, *Ipomoea batatas* (L.) Lam, exhibit self- and cross-incompatibility, which leads to low seed set (Fujise 1964, Martin 1965). This low seed set poses a remarkable challenge to achieving efficient expansion of genetic variation through hybridization in sweet potato breeding. Many investigations have been conducted to determine the factors responsible for low seed set, including those by Wada (1923), Terao (1934), Togari and Kawahara (1946a, 1946b), Shigemura (1943), Hernandez and Miller (1962, 1964), Fujise (1964), Wang (1964), Martin (1965, 1968), Martin and Ortiz (1965, 1967), and Martin and Cabanillas (1966). Togari and Kawahara (1946a, 1946b) reported the association of pollen germination rate and growth rates of pollen tubes with the rate of capsule setting and the number of seeds per capsule among different compatible crossings. Ting and Kehr (1953) reported that meiotic abnormalities may be responsible for low seed set in some sweet potato lines. However, Jones (1965), who was unable to associate meiotic abnormalities with seed set, suggested that the low seed set observed in sweet potatoes is generally due to causes other than meiotic abnormalities, such as diseases, genetically controlled incompatibility, sterility, or physiological dysfunction in developing seed.

In a prior study, the author reported that most embryo sacs that had not been penetrated by pollen tubes and were evaluated 48 hours after pollination exhibited abnormalities, such as the interruption of the egg apparatus development halfway. Conversely, the number of normal young embryos was closely associated with the number of seeds per capsule, and no embryonic development abnormalities were observed (Kokubu *et al.* 1982, Murata and Matsuda 2003). These findings suggest that an abundance of abnormal embryo sacs may be the main cause of low seed set in sweet potatoes.

*Ipomoea trifida* (H. B. K.) DON is a closely related species to sweet potatoes and forms a polyploid complex series consisting of diploid (2n = 30), tetraploid (2n = 60), and hexaploid (2n = 90) lines. In potted cultivation after grafting to morning glory, diploids produced more than 1,500 flowers per pot, whereas tetraploids and hexaploids produced 400–600 and 150–200 flowers, respectively. Moreover, the fertility rate [Number of fertile seeds/ Number of crossed flowers × 4) × 100] of diploids was approximately 90%, while it was 40–60% for tetraploids and 20–30% for hexaploids [K. A. E. S. (Kyushu Agr. Exp. Sta. Ibusuki, Kagoshima)] (1971–1985). This study aims to elucidate the relationship between low seed set and abnormal embryo sacs in sweet potatoes by comparing diploid, tetraploid, and hexaploid lines of *I. trifida* with sweet potato cultivars and lines. Additionally, the study seeks to explore the evolutionary underpinnings of the high frequency of abnormal embryo sacs in sweet potatoes.

## Materials and Methods

This experiment utilized *I. trifida* diploid (2n = 30), tetraploid (2n = 60), and hexaploid (2n = 90) lines, as well as sweet potato cultivars and lines (Table 1). To induce flowering, the stems were grafted onto the dwarf type morning glory (*Ipomoea nil* cv. ‘Kidachi’). The grafted plants were cultivated in a greenhouse with a temperature range of 18°C to 30°C, which is known to be optimal for sweet potato seed set (Fujise 1964, Wang 1964). On the day of flowering, ovaries were collected between 9 and 10 a.m. The collected ovaries were fixed with F.A.A. fluid (a mixture of 50% ethanol, formalin, and acetic acid at a ratio of 90:5:5) and then cut transversely or longitudinally into 15 μm thick sections by the ordinary paraffin method. The sections were then stained with Safranin O and Fast green.

Data were analyzed statistically using Tukey-Kramer’s multiple test in JMP11 software (SAS). The mean number of normal and abnormal embryo sacs in *I. trifida* were compared using protected least significant differences (LSD) at 5% level.

## Results

### Number of ovules per ovary

**Fig. 1.**
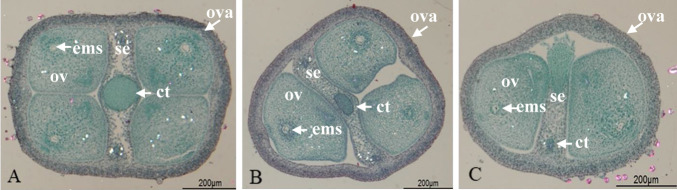
Ovarian transverse sections. A: Four ovules per ovary, B: Three ovules per ovary, C: Two ovules per ovary. ct; conducting tissue, ems; embryo sac, ov; ovule, ova; ovary, se; septum.

The number of ovules per ovary is presented in Table 1. In the transverse section, there were usually four ovules per ovary, divided into two ovules by a septum with an oval-shaped induction tissue in the center (Fig. 1A). In *I. trifida*, the diploid and tetraploid lines had four ovules per ovary, whereas the hexaploid lines had a higher number of ovules than the four normal lines, with three out of 17 ovaries having five ovules and one having six ovules per ovary.

**Fig. 2.**
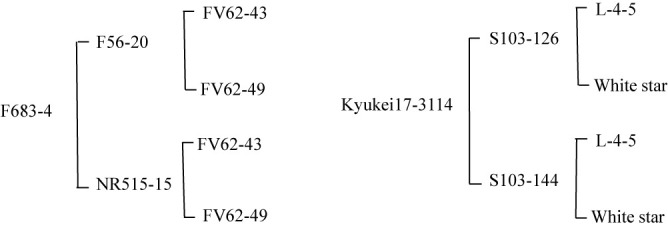
Pedigrees of two sib-cross lines.

In sweet potatoes, most cultivars and lines had four ovules per ovary, but only ‘Choshu’ and ‘Minamiyutaka’ had three ovules in two of the 20 ovaries observed. However, the frequency of abnormal numbers of ovules was particularly high in the sib-cross lines ‘F683-4’ and ‘Kyukei 17-3114’ of foreign cultivars, with 19 out of 84 ovaries examined (22.6%) in ‘F683-4’ and four out of 18 ovaries examined (22.2%) in ‘Kyukei 17-3114’ having two or three ovules (Fig. 1B, 1C). The pedigree of two sib-cross lines is shown in Fig. 2. ‘F683-4’ is a Brazilian sib-cross line (inbreeding coefficient: 25.0), and ‘Kykei 17-3114’ is an American sib-cross line (inbreeding coefficient: 25.0), both of which are inbred lines by sib-cross.

### Number of normal embryo sacs per ovary

**Fig. 3.**
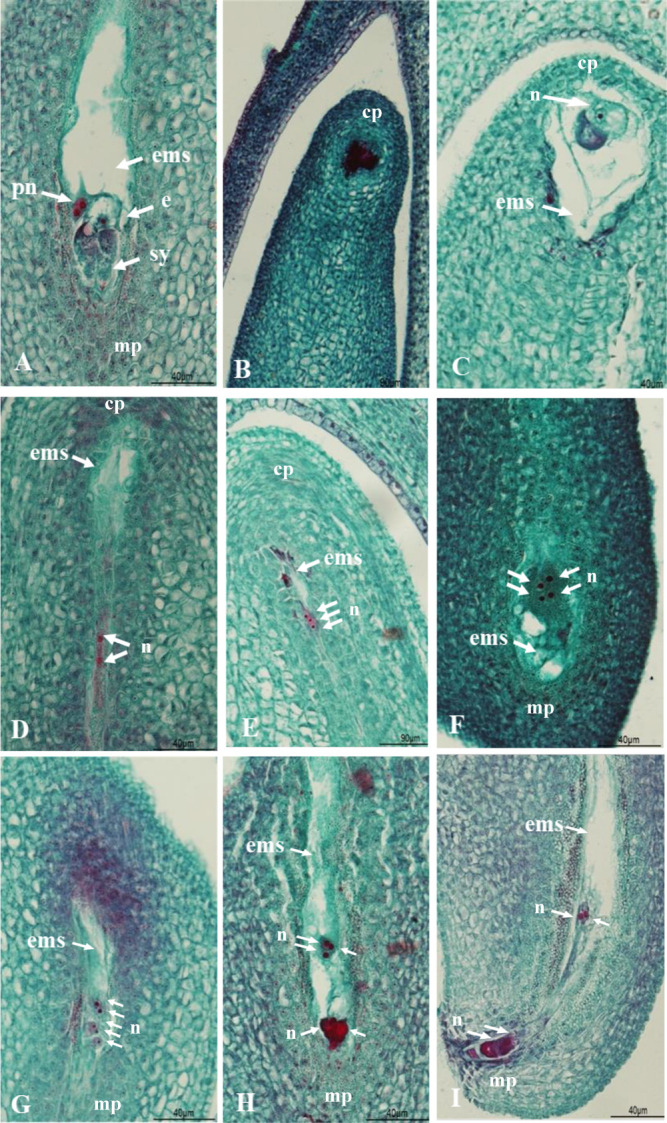
Longitudinal sections of normal and abnormal embryo sacs. A: Normal embryo sac comprised of one egg cell, two assistant cells, and two polar nuclei. B and C: abnormal embryo sacs (A type) exhibited halted division at the uninuclear stage. D, E, and F: abnormal embryo sacs (B type) exhibited halted division at the two or four nuclear stages. G, H, and I: abnormal embryo sacs (C type) exhibited halted division after the four nuclear stages. cp; chalaza part, e; egg cell, ems; embryo sac, mp; micropyle side, n; nucleus, pn; polar nuclei, sy; synergid.

Normal embryo sacs consisted of one egg cell, two assistant cells, and two polar nuclei. (Fig. 3A). In contrast, the other embryo sacs were considered abnormal (Fig. 3B–3I). The numbers of normal embryo sacs per ovary in *I. trifida* are shown in Table 2. In the two diploid lines of *I. trifida*, most of the embryo sac observed were normal, with very few abnormal embryo sac. The average number of normal embryo sacs per ovary was 3.93 in the diploid, 3.55 in the tetraploid and 2.53 in the hexaploid lines, thus there was a significant decrease in normal embryo sacs with increasing ploidy.

The number of normal embryo sac per ovary in the sweet potato cultivars and lines is shown in Table 3. The number of normal embryo sacs per embryo was less than one in ‘Hi-Starch’and ‘Tokimasari’, at 0.63 and 0.60, respectively. Conversely, ‘Koganesengan’ and ‘Benikomachi’ had more than three normal embryo sacs per ovule, 3.20 and 3.72, respectively. In sweet potatoes, the frequency of normal embryo sac varied greatly among cultivars and lines. Out of 437 ovaries sampled from 29 cultivars and lines, 13.0% had normal embryo sacs in all four ovules, and the mean number of normal embryo sac per ovary was 1.96.

### Percentage and variation of abnormal embryo sac

To identify the stage at which abnormal embryo sac occurs during female gametogenesis, it was classified into three types: A, B, and C.

A: The chalaza parts were stained dark red with Safranin O (Fig. 3B) or in a uninucleate stage (Fig. 3C) due to early-stage division arrest in female gametogenesis.

B: Division in female gametogenesis ceased, dividing it into two or four nuclear stages (Fig. 3D–3F).

C: Female gametogenesis stops dividing into four to eight nuclear stages.

Type C abnormal embryo sacs were identified, characterized by the absence of cell membranes or halted in the egg cells and synergids, specifically on the micropyle side (Fig. 3G–3I).

The percentages and variations of abnormal embryo sac in *I. trifida* are shown in Table 4, whereas those in sweet potato cultivars and lines are demonstrated in Table 5. The frequency of abnormal embryo sacs in *I. trifida* increased with increasing polyploidy, and the mean number of abnormal embryo sacs per ovary was 0.07 in the diploid, 0.45 in the tetraploid and 1.47 in the hexaploid lines, i.e., also significantly increasing with ploidy. Almost all embryo sacs in the diploid lines were normal, whereas abnormal embryo sac were observed in the tetraploid and hexaploid lines, with the majority being type A and division stopping at an early stage in female gametogenesis. In sweet potatoes, although there were variations in the type of abnormal embryo sacs depending on the cultivars and lines, variations of abnormal embryo sacs were observed in the following order: Type A (51.8%), Type C (30.5%), and Type B (17.7%) (Table 5).

The relationship between the frequency of abnormal embryo sac and polyploidy is presented in Table 6, demonstrating that on diploid *I. trifida* plants, the frequency of abnormal embryo sac was 2.5%, whereas tetraploid and hexaploid plants had frequencies of 11.3% and 37.0%, respectively. This indicates that the frequency of abnormal embryo sacs increased with increasing ploidy in *I. trifida*. In terms of variation of abnormal embryo sacs, tetraploid and hexaploid lines of *I. trifida* and sweet potato cultivars and lines showed similar frequencies of expression for type A at 58.3%, 59.3%, and 51.8%, respectively.

## Discussion

Histological observation of sweet potato ovaries on the flowering day revealed the presence of many abnormal embryo sacs devoid of typical female structures, including one egg cell, two assistant cells, and two polar nuclei. These abnormalities largely contribute to the corresponding low seed set. Studies by Fujise *et al.* (1957), Fujise (1964), and Yunoue and Hirosaki (1974) have shown that if heterozygosity is reduced by autogamy or inbreeding such as sib-cross, inbreeding depression appears strongly in the flower organs, particularly in the male organs. Our findings, as shown in Table 1, suggest that inbreeding depression is also strongly manifested in female organs, as evidenced by the large numbers of two and three ovules per ovary observed in the sib-cross lines. Burnham (1967) observed the number of ovules per ovary in 30 lines and found that 25 lines had normal four ovules, whereas five lines had three or fewer, and reported that in lines such as the latter, a reduced ovule number was the cause of low seed set. In the present study, no ovaries with fewer than four ovules were observed in any of the lines, regardless of ploidy, in *I. trifida*, except for two sib-cross lines in sweet potato. In most of the ovaries, all four ovules were present, indicating that the number of ovules per ovary was not reduced. Therefore, the reduced number of ovules per ovary does not seem to be directly responsible for the low seed set in these cultivars and lines. However, in the two sib-cross lines with a reduced number of ovules per ovary, the reduced number of ovules was likely one of the causes of low seed sets, as reported by Burnham (1967). This suggests that breeding by crossing closely related lines is undesirable for fertility.

Because normal embryo sacs are expected to form seeds if pollination, fertilization, and embryogenesis are normal, we initially examined the frequency of normal embryo sacs in each ovary on the flowering day for each *I. trifida* and sweet potato cultivar and line. The K. A. E. S. (Kyushu Agr. Exp. Sta. Ibusuki, Kagoshima) (1963) reported that the relationship between high and low fertility among cross combinations was nearly consistent from year to year, suggesting a genetic basis for fertility. On the other hand, Fujise (1964) reported the high temperatures (above 34–36°C) or low temperatures (below 14–16°C) may affect seed set. Since the present experiment was conducted under the optimal conditions for sampling as described above, the relationship between temperature and the number of normal embryo sacs should be further verified in the future. Therefore, the observed differences in the number of normal embryo sac among sweet potato cultivars and lines in this study may be attributed to genetic characteristics concerning fertility.

In a previous study, Murata (1986) reported that embryo sac mother cells undergo meiosis 9–10 days before flowering, while antipodal cells degenerate 1–3 days before flowering. Moreover, a normal embryo sac on the flowering day has one egg cell, two assistant cells, and two polar nuclei. To elucidate the stage at which abnormal embryo sac occur during female gametogenesis, they were classified into three types. In *I. trifida*, the mean number of abnormal embryo sacs per ovary significantly increased with increasing ploidy (Table 4). More than half of the *I. trifida* and sweet potato cultivars and lines exhibited abnormal embryo sacs classified as type A, which cease development at an early stage. These results indicated that meiotic defects in embryo sac mother cells contribute to the development of abnormal embryo sacs in sweet potatoes. Ting and Kehr (1953) identified meiotic abnormalities in pollen mother cells as the cause of low fertility in sweet potatoes. Likewise, Wang (1964) reported that aberrant division of chromosomes during meiosis leads to the production of large amounts of sterile pollen. In contrast, Jones (1965) observed normal meiosis in pollen mother cells of 10 lines and attributed low fertility to genetic sterility, self-incompatibility, physiological imbalance in seed development, or bud disease resulting from infection. While most previous reports have attributed low seed set to abnormal pollen meiosis, morphological observations have been limited, and no study has specifically examined the cause of abnormal female gametogenesis, as demonstrated by this experiment.

Hybridization trials have been conducted in Japan by grafting sweet potatoes onto morning glory rootstocks to promote flowering, followed by artificial crosses after flowering. Martin and Jones (1971) conducted a study on fertility in Puerto Rico, located in the Caribbean Sea, in which several generations of random mating were performed. The results showed no tendency for the fertility rate to increase after seven generations, indicating that sweet potato is a low-fertility plant, even under natural conditions in the tropics. This suggests that creating selection pressure for low fertility by advancing generations under natural conditions is a strenuous process.

In this study, the author concluded that the main cause of low fertility in sweet potatoes is the high frequency of abnormal embryo sacs, which is presumably due to a defect in the meiosis of the embryonic mother cell. While genes regulating abnormal meiosis are identified in various plant species [e.g., synaptic mutant genes that disrupt synapse and chiasma formation in barley, tomato, and maize (Kaul and Murthy 1985)], no such defective genes have been identified in sweet potato. This could be attributed, in part, to sweet potato being a higher-order hexaploidy.

Shiotani and Kawase (1980, 1989) reported, through cytogenetic studies, that sweet potatoes are autohexaploid. Furthermore, Shiotani and Kawase (1981), in contrasting the general characteristics of a closely related wild species (*I. trifida*) and the cultivated sweet potato, speculated that the observed sterility in sweet potatoes may be closely related to the formation of polyvalent chromosomes that are associated with autohexaploidy. Based on these findings, the high frequency of abnormal embryo sac, which was found to be a major cause of low seed set in sweet potatoes in this study, is likely closely related to their autohexaploidy. In line with the findings of this study, the author speculated that the transition from the closely related wild species (*I. trifida*) with seed production to sweet potato, which propagates vegetatively, may have led to abnormalities in female gametogenesis as sweet potato became less dependent on seed reproduction.

## Author Contribution Statement

T.M. conceived and designed the experiments. T.M. performed the experiments, analyzed the data, and wrote the manuscript.

## Figures and Tables

**Table 1.** Number of ovules per ovary in *Ipomoea* *trifida* closely related to sweet potato and sweet potato cultivars and lines

Species name	Group and Norin No.	Cultivar and line	Polyploidy	Number of ovules per ovary					Number of ovaires examined
				2	3	4	5	6	
*Ipomoea trifida*	*Ipomoea* species closely related to sweet potato	K450	2x			20			20
		K221				10			10
		K500-1	4x			16			16
		K300-1				20			20
		K233-1				17			17
		K123-11	6x			13	3	1	17
Sweeet potato (*Ipomoea batatas*)	Breeding line	Chikei 682-11	6x			19			19
		Kyushu No. 58				19			19
	Domestic cultivar	Shichifuku	6x			20			20
		Choshu			2	18			20
		Tsurunashigenji				13			13
	31	Koganesenngan	6x			20			20
	33	Minamiyutaka			2	18			20
	34	Benikomachi				18			18
	36	Beniazuma				20			20
	38	Shiroyutaka				16			16
	39	Shirosatsuma				10			10
	40	Satsumahikari				6			6
	41	Hi-Starch				12			12
	43	Beniotome				7			7
	44	Hitachired				21			21
	46	Joywhite				4			4
	47	Ayamurasaki				27			27
	54	Murasakimasari				58			58
	55	Benimasari				11			11
	56	Purple Sweet Lord				15			15
	59	Daichinoyume				9			9
	61	Okikogane				3			3
	62	Akemurasaki				15			15
	63	Tokimasari				10			10
	64	Beniharuka				11			11
	Sib-cross line derived from foreign line	F683-4	6X	12	7	65			84
		Kyukei17-3114		2	2	14			18
	Line derived from foreign line	FV62-41	6x	1		19			20
		Kyukei17-3104				19			19

**Table 2.** Number of normal embryo sacs per ovary in *Ipomoea trifida* closely related to sweet potato

Line No.	Polyploidy	Number of normal embryo sacs per ovary (%)					Number of ovaires examined	Mean number of normal embryo sacs per ovary
		0	1	2	3	4		
K450	2x	0	0	0	1	19	20	3.95
					(5.0)	(95.0)		
K221		0	0	0	1	9	10	3.90
					(10.0)	(90.0)		
Total		0	0	0	2	28	30	3.93^a^
					(6.7)	(93.3)		
K500-1	4x	0	0	2	7	7	16	3.31
				(12.4)	(43.8)	(43.8)		
K300-1		0	0	2	4	14	20	3.60
				(10.0)	(20.0)	(70.0)		
K233-1		0	0	0	5	12	17	3.71
					(29.4)	(70.6)		
Total		0	0	4	16	33	53	3.55^b^
				(7.5)	(30.2)	(62.3)		
K123-11	6x	0	3	6	4	4	17	2.53^c^
			(17.6)	(35.4)	(23.5)	(23.5)		

Values followed by the same letter are not significantly different at 5% by Tukey-Kramer’s multiple test.

**Table 3.** Number of normal embryo sacs per ovary in sweet potato cultivars and lines

Group and Norin No.	Cultivar and line	Number of normal embryo sacs per ovary (%)					Number of ovaires examined	Mean number of normal embryo sacs per ovary
		0	1	2	3	4		
Breeding line	Chikei 682-11	1	5	5	4	4	19	2.26
		(5.2)	(26.3)	(26.3)	(21.1)	(21.1)		
	Kyushu No.58	0	2	6	7	4	19	2.68
			(10.5)	(31.6)	(36.8)	(21.1)		
Domestic cultivar	Shichifuku	3	7	3	6	1	20	1.75
		(15.0)	(35.0)	(15.0)	(30.0)	(5.0)		
	Choshu	0	6	1	4	1	12	2.00
			(15.0)	(8.3)	(33.4)	(8.3)		
	Tsurunashigenji	2	8	3	0	0	13	1.08
		(15.4)	(61.5)	(23.1)				
31	Koganesengan	0	0	4	8	8	20	3.20
				(20.0)	(40.0)	(40.0)		
33	Benikomachi	0	0	1	3	14	18	3.72
				(5.6)	(16.7)	(77.7)		
34	Minamiyutaka	0	2	10	6	2	20	2.40
			(10.0)	(50.0)	(30.0)	(10.0)		
36	Beniazuma	3	6	6	4	1	20	1.70
		(15.0)	(30.0)	(30.0)	(20.0)	(5.0)		
38	Shiroyutaka	1	6	7	0	0	14	1.42
		(7.1)	(42.9)	(50.0)				
39	Shirosatsuma	2	1	1	1	0	5	1.20
		(40.0)	(20.0)	(20.0)	(20.0)			
40	Satsumahikari	1	3	0	0	1	5	1.40
		(20.0)	(60.0)			(20.0)		
41	Hi-Starch	4	3	1	0	0	8	0.63
		(50.0)	(37.5)	(22.5)				
43	Beniotome	0	4	7	4	1	16	2.13
			(25.0)	(43.8)	(25.0)	(6.2)		
44	Hitachired	2	4	3	0	0	9	1.11
		(22.2)	(44.5)	(33.3)				
46	Joywhite	0	0	4	2	1	7	2.57
				(57.1)	(28.6)	(14.3)		
47	Ayamurasaki	1	7	8	4	2	22	1.87
		(4.5)	(31.8)	(36.5)	(18.1)	(9.1)		
54	Murasakimasari	12	19	11	6	3	51	1.39
		(23.5)	(37.2)	(21.6)	(11.8)	(5.9)		
55	Benimasari	2	3	5	1	0	11	1.45
		(18.1)	(27.2)	(45.6)	(9.1)			
56	Purple Sweet Lord	2	2	3	5	1	13	2.07
		(15.4)	(15.4)	(23.1)	(38.4)	(7.7)		
59	Daichinoyume	1	1	0	0	0	2	1.00
		(50.0)	(50.0)					
61	Okikogane	0	1	0	2	0	3	2.33
			(33.3)		(66.7)			
62	Akemurasaki	5	6	2	2	0	15	1.06
		(33.4)	(40.0)	(13.3)	(13.3)			
63	Tokimasari	2	3	0	0	0	5	0.60
		(40.0)	(60.0)					
64	Beniharuka	0	2	2	6	1	11	2.54
			(18.2)	(18.2)	(54.6)	(9.0)		
Sib-cross line derived from foreign line	FV62-41	6	5	4	5	0	20	1.40
		(30.0)	(25.0)	(20.0)	(25.0)			
	Kyukei 17-3114	2	2	2	6	6	18	2.67
		(11.1)	(11.1)	(11.1)	(33.3)	(33.3)		
Line derived from foreign line	F683-4	2	5	9	4	2	22	1.95
		(9.1)	(22.7)	(40.9)	(18.2)	(9.1)		
	Kyukei 17-3104	2	2	4	7	4	19	2.47
		(12.5)	(10.5)	(21.1)	(36.8)	(21.1)		
	Total	56	115	112	97	57	437	1.96
	(%)	(12.9)	(26.3)	(25.6)	(22.2)	(13.0)		

**Table 4.** Percentage and variation of abnormal embryro sacs in *Ipomoea trifida* closely related to sweet potato

Line No.	Polyploidy	Number of ovaries examined	Number of embryo sacs examined	Number of normal embryo sacs	Number of abnormal embryo sacs	% of abnormal embryo sacs	Variation of abnormal embryo sac*^a^* (%)			Mean number of abnormal embryo sacs per ovary
							A	B	C	
K450	2x	20	80	78	2	2.5	0	0	2 (100)	0.05
K221		10	40	39	1	2.5	1 (100)	0	0	0.10
Total		30	120	117	3	2.5	1 (33.3)	0	2 (66.7)	0.07^c^
K500-1	4x	16	64	53	11	17.2	7 (63.6)	4 (36.4)	0	0.69
K300-1		20	80	72	8	10.0	4 (50.2)	2 (25.0)	2 (25.0)	0.40
K233-1		17	68	63	5	7.4	3 (60.0)	1 (20.0)	1 (20.0)	0.29
Total		53	212	188	24	11.3	14 (58.3)	7 (29.2)	3 (12.5)	0.45^b^
K123-11	6x	19	73	46	27	37.0	16 (59.3)	3 (11.1)	8 (29.6)	1.47^a^

Values followed by the same letter are not significantly different at 5% by Tukey-Kramer’s multiple test. *^a^* A: Abnormal embryo sac which stopped to divide at the uninuclear stage. B: Abnormal embryo sac which stopped to divide at the two or four nuclear stage. C: Abnormal embryo sac which stopped to divide after the four nuclear stage.

**Table 5.** Percentage and variation of abnormal embryro sacs in sweet potato cultivars and lines

Group and Norin No.	Cultivar and line	Number of ovaries examined	Number of embryo sacs examined	Number of normal embryo sacs	Number of abnormal embryo sacs	% of abnormal embryo sacs	Variation of abnormal embryo sac*^a^* (%)			Mean number of abnormal embryo sacs per ovary
							A	B	C	
Breeding line	Chikei 682-11	19	76	43	33	43.2	8 (24.2)	14 (42.4)	11 (33.3)	1.74
	Kyushu No.58	19	76	51	25	32.9	3 (12.0)	9 (36.0)	13 (52.0)	1.32
Domestic cultivar	Shichifuku	20	80	35	45	56.3	8 (17.8)	16 (35.6)	21 (46.7)	2.25
	Choshu	20	78	40	38	48.7	12 (31.6)	8 (21.1)	18 (47.4)	1.90
	Tsurunashigenji	13	52	14	38	73.1	9 (23.7)	7 (18.4)	22 (57.9)	2.92
31	Koganesengan	20	80	64	16	20.0	4 (25.0)	1 (6.3)	11 (68.8)	0.80
33	Benikomachi	18	72	67	5	6.9	2 (40.0)	2 (40.0)	1 (20.0)	0.28
34	Minamiyutaka	20	78	49	29	37.2	6 (20.7)	9 (31.0)	14 (48.3)	1.45
36	Beniazuma	20	80	34	46	57.5	18 (39.1)	1 (2.2)	27 (58.7)	2.30
38	Shiroyutaka	16	64	20	44	68.7	35 (79.5)	4 (9.1)	5 (11.4)	2.75
39	Shirosatsuma	10	44	12	32	80.0	17 (53.1)	3 (9.4)	12 (37.5)	3.20
40	Satsumahikari	6	24	9	15	62.5	9 (60.0)	4 (26.7)	2 (13.3)	2.50
41	Hi-Starch	12	48	5	43	89.6	30 (69.8)	7 (16.3)	6 (14.0)	3.58
43	Beniotome	7	28	18	10	35.7	4 (40.0)	0	6 (60.0)	1.43
44	Hitachired	21	84	24	60	71.4	42 (70.0)	10 (16.7)	8 (13.3)	2.86
46	Joywhite	4	16	12	4	25.0	0	1 (25.0)	3 (75.0)	1.00
47	Ayamurasaki	27	108	50	58	53.7	34 (58.6)	6 (10.3)	18 (31.0)	2.15
54	Murasakimasari	58	232	79	153	65.9	120 (78.4)	16 (10.5)	17 (11.1)	2.64
55	Benimasari	11	44	16	28	63.6	13 (46.4)	9 (32.1)	6 (21.4)	2.55
56	Purple Sweet Lord	15	60	30	30	50.0	13 (43.3)	2 (6.7)	15 (50.0)	2.00
59	Daichinoyume	9	36	13	23	63.9	12 (52.2)	4 (17.4)	7 (30.4)	2.56
61	Okikogane	3	12	7	5	41.7	2 (40.0)	0	3 (60.0)	1.67
62	Akemurasaki	15	60	16	44	73.3	34 (77.3)	4 (9.1)	6 (13.6)	2.93
63	Tokimasari	10	20	2	18	90.0	15 (83.3)	1 (5.6)	2 (11.1)	1.80
64	Beniharuka	11	44	28	16	35.5	8 (50.0)	0	8 (50.0)	1.45
Sib-cross line derived from foreign line	F683-4	20	78	43	35	44.9	11 (31.4)	11 (31.4)	13 (37.1)	1.75
	Kyukei17-3114	17	66	48	18	27.3	4 (22.2)	9 (50.0)	5 (27.8)	1.06
Line derived from foreign line	FV62-41	20	78	28	50	64.1	29 (58.0)	7 (14.0)	14 (28.0)	2.50
	Kyukei17-3104	19	76	47	29	38.2	11 (37.9)	10 (34.5)	8 (27.6)	1.53
	Total	404	1894	904	990	52.3	513 (51.8)	175 (17.7)	302 (30.5)	2.45

*^a^* A: Abnormal embryo sac which stopped to divide at the uninuclear stage. B: Abnormal embryo sac which stopped to divide at the two or four nuclear stage. C: Abnormal embryo sac which stopped to divide after the four nuclear stage.

**Table 6.** Relationship between the frequency of abnormal embryro sacs and polyploidy

Group	Polyploidy	Number of ovaries examined	Number of embryo sacs examined	Number of normal embryo sacs	Number of abnormal embryo sacs	% of abnormal embryo sacs	Variation of abnormal embryo sac*^a^* (%)		
							A	B	C
*Ipomoea trifida*	2x	30	120	117	3	2.5	1	0	2
							(33.3)		(66.7)
	4x	53	212	188	24	11.3	14	7	3
							(58.3)	(29.2)	(12.5)
	6x	19	73	46	27	37.0	16	3	8
							(59.3)	(11.1)	(29.6)
Sweet potato (*Ipomoea batatas*)	6x	404	1894	904	990	52.3	513	175	302
							(51.8)	(17.7)	(30.5)

*^a^* A: Abnormal embryo sac which stopped to divide at the uninuclear stage. B: Abnormal embryo sac which stopped to divide at the two or four nuclear stage. C: Abnormal embryo sac which stopped to divide after the four nuclear stage.
